# The endocannabinoid systemʼs involvement in motor development relies on cannabinoid receptors, TRP channels, and Sonic Hedgehog signaling

**DOI:** 10.14814/phy2.15565

**Published:** 2023-01-12

**Authors:** Lakhan S. Khara, Declan W. Ali

**Affiliations:** ^1^ Department of Biological Sciences University of Alberta Edmonton Edmonton Alberta Canada; ^2^ Department of Physiology University of Alberta Edmonton Edmonton Alberta Canada; ^3^ The Neuroscience and Mental Health Institute University of Alberta Edmonton Edmonton Alberta Canada

**Keywords:** behavior, cannabinoid, loco, motor, motor, sensory

## Abstract

The endocannabinoid system (eCS) plays critical roles in locomotor function and motor development; however, the roles of non‐canonical cannabinoid receptor systems such as transient receptor potential (TRP) channels and the Sonic Hedgehog (SHH) signaling pathway in conjunction with the eCS in sensorimotor development remains enigmatic. To investigate the involvement of canonical and non‐canonical cannabinoid receptors, TRP channels, and the SHH pathway in the development of sensorimotor function in zebrafish, we treated developing animals with pharmacological inhibitors of the CB1R, CB2R, TRPA1/TRPV1/TRPM8, and a smoothened (SMO) agonist, along with inhibitors of the eCS catabolic enzymes fatty acid amide hydrolase (FAAH) and monoacylglycerol lipase (MAGL) during the first ~24 h of zebrafish embryogenesis. Locomotor function was examined by assessing touch‐evoked escape swimming at 2 days post‐fertilization. We report that FAAH inhibition had no effect on swimming while MAGL inhibition using JZL 184 reduced swimming distance and the dual FAAH/MAGL inhibitor JZL 195 impaired swimming distance and mean swimming velocity. The CB1R antagonist AM 251 prevented locomotor deficits caused by eCS perturbation but the CB2R antagonist AM 630 did not. Inhibition of TRPA1/TRPV1/TRPM8 using AMG 9090 rescued the locomotor reductions caused by FAAH/MAGL inhibition, but not by MAGL inhibition alone. The SMO agonist purmorphamine attenuated the effects of JZL 184 and JZL 195 on swimming distance, but not mean velocity. Together, these findings provide one of the first investigations examining the interactions between the eCS and its non‐canonical receptor systems in vertebrate motor development.

## Abstract 2

Perturbation of the endocannabinoid system in developing zebrafish by application of FAAH and MAGL alters sensorimotor activity. These effects are partially prevented by CB1R and TRP antagonists, but not CB2R blockers.

## 1 INTRODUCTION

The endocannabinoid system (eCS) plays a developmental role in several neurobiological processes such as motor and sensory function, axonal growth, and synaptic development (Guindon & Hohmann, 2009; Kano et al., 2009; Martella et al., 2016; Watson et al., 2008). The endocannabinoids (eCBs) anandamide (AEA) and 2‐arachidonoylglycerol (2‐AG) act as the signaling molecules within the eCS and are tightly regulated by the enzymes N‐acyl‐phosphatidylethanolamine phospholipase D (NAPE‐PLD) and diacylglycerol lipase (DAGL), which mediate the synthesis of AEA and 2‐AG, respectively, along with the catabolic enzymes fatty acid amide hydrolase (FAAH) and monoacylglycerol lipase (MAGL), which break down AEA and 2‐AG, respectively (Lu & Mackie, 2016). eCB signaling can be perturbed by blocking the activity of FAAH and MAGL, leading to over‐activation of the eCS (Gobbi et al., 2005; Griebel et al., 2015; Long et al., 2009). Inhibiting the enzymes FAAH and MAGL has been shown to decrease locomotion and alter motor neuron development (Boa‐Amponsem et al., 2019; Seillier et al., 2014; Sufian et al., 2021). Furthermore, we have recently observed that FAAH/MAGL inhibition alters functional locomotor and sensorimotor development in zebrafish (Khara et al., 2022). These previous findings highlight that endocannabinoid signaling is critical in motor function, however, the mechanisms involved require further examination.

The most well‐described mechanism of cannabinoid signaling is through the cannabinoid receptors: CB1R and CB2R, as these are the canonical cannabinoid receptors with affinity for eCBs and plant‐derived phytocannabinoids (Mackie, 2005). These cannabinoid receptors are inhibitory G‐protein‐coupled receptors whose activation leads to a reduction of intracellular cAMP by inhibiting the activity of adenylyl cyclase to modulate synaptic activity (Howlett, 2002). Studies in mammalian and non‐mammalian vertebrate models support that CB1R is crucial in the motor development aspects of the eCS (Drews et al., 2005; Luchtenburg et al., 2019; Sufian et al., 2021; Watson et al., 2008). However, cannabinoids interact with receptors other than CB1R and CB2R and thus motor development may be impacted through the interactions of eCBs with non‐canonical cannabinoid receptor systems. For instance, eCBs, phytocannabinoids, and synthetic cannabinoid receptor agonists have affinity for the transient receptor potential (TRP) family of cation channels, where a variety of different TRP subtypes are suggested to operate as ionotropic cannabinoid receptors (Morales et al., 2017; Muller et al., 2019). Even though TRP channels are well‐known for their involvement in sensory systems, the exact nature of their interaction with cannabinoids and whether this interplay contributes to motor development is largely unknown.

Recent studies have found that eCBs interact with the Sonic Hedgehog (SHH) signaling pathway by suppressing the transmembrane protein Smoothened (SMO), leading to downregulated SHH signaling (Khaliullina et al., 2015). In zebrafish, cannabinoid‐induced microphthalmia can be rescued with *Shh* mRNA injections or by treatment with CB1R antagonists, (Fish et al., 2019) while another study found that *Shh* mRNA injection prevented behavioral and locomotor alterations caused by inhibition of FAAH and MAGL (Boa‐Amponsem et al., 2019). These findings provide evidence for the interplay between the eCS and SHH signaling, but how these interactions underlie motor development is unknown.

Elucidating these alternative mechanisms is crucial for our fundamental understanding of the eCS and how it contributes to neurobiological development. Thus, given that the current information pertaining to alternative mechanisms of eCB signaling is limited, we sought to identify whether these non‐canonical receptor systems and signaling mechanisms are involved in mediating the motor deficits caused by eCS perturbation in zebrafish embryos. We have previously demonstrated that singular FAAH inhibition does not alter escape swimming, but that singular inhibition of MAGL and simultaneous inhibition of FAAH and MAGL causes significant deficits to escape swimming (Khara et al., 2022). Thus, our goal in this study was to treat embryonic zebrafish with eCB degradation inhibitors and test whether cannabinoid receptor antagonists or SHH agonists could alter the effects of the inhibitors.

## 2 MATERIALS AND METHODS

### 2.1 Animal care, drugs, and exposure

The animals used in this study were wild‐type zebrafish (*Danio rerio*) of the Tubingen Longfin (TL) strain. All animal housing and experimental procedures in this study were approved by the Animal Care and Use Committee of the University of Alberta (AUP #00000816) and was in compliance with the Canadian Council on Animal Care guidelines of the humane use of animals for research purposes. For breeding and egg collection, adult male and female zebrafish were placed in breeding tanks on the evening before eggs were required. The following morning, eggs were collected immediately after fertilization. Embryos and larvae were housed in a 28.5°C incubator set on a 12‐h light/dark cycle. Embryos and larvae were provided with embryo media (60 mg/ml Instant Ocean, pH 7.0).

The pharmacological compounds used in this study are the selective FAAH inhibitor URB 597, the selective MAGL inhibitor JZL 184, and the dual FAAH/MAGL inhibitor JZL 195 (all obtained from Adooq Bioscience). URB 597 and JZL 195 were tested at the 5 μM concentration, while JZL 195 was tested at the 2 μM concentration. Along with these compounds, animals were co‐treated with the CB1R antagonist AM 251 (Selleck Chemicals), the CB2R antagonist AM630 (Adooq Bioscience), the TRPV1/TRPA1/TRPM8 inhibitor AMG 9090 (Alomone Labs), or the SMO agonist Purmorphamine (Sigma‐Aldrich). The concentrations used for these four drugs were 10 nM for AM 251, 1 μM for AM 630, 0.01 μM for AMG 9090, and 5 μM for purmorphamine and were determined based on previous studies (Khara et al., 2022; Sufian et al., 2021) and dose–response relationships performed for survival, hatching, morphology, and locomotion in embryos and larva. All compounds listed here were dissolved in dimethyl sulfoxide (DMSO), which was used as the vehicle control. The drug exposure took place immediately after egg fertilization until 24 h post‐fertilization (hpf), effectively spanning from ~0–24 hpf as done previously (Khara et al., 2022; Sufian et al., 2021). Embryos were exposed to the compounds via the embryo media. At 24 hpf, all exposure media was washed‐out and replaced with fresh embryo media. Following the exposure period, animals were allowed to develop until needed at 2 days post‐fertilization (dpf).

### 2.2 Escape swimming assessments

To assess escape swimming following a C‐start escape response, individual 2 dpf embryos were positioned in the center of a 140 mm petri dish containing embryo media. The petri dish was set on top of an infrared backlight source to be viewed by a Basler GenICam scanning camera (Basler acA1300‐60gm) with a 75 mm f2.8 C‐mount lens, provided by Noldus. Escape swimming in zebrafish embryos was triggered by delivering an acute mechanical stimulus to the head of the embryo by using a thin fishing line (Berkley Fishing; model # BGQS60C‐15). Each embryo was tested alone and swimming performance was analyzed using the movement tracking software EthoVision XT‐11.5 (Noldus). Mean swimming distance (cm) and mean swimming velocity (cm/s) were analyzed as metrics of locomotion when assessing escape swimming performance.

### 2.3 Statistics

All statistical analyses were performed using GraphPad Prism (version 9.2.0). Normality of data was examined using the Shapiro–Wilk test. The data presented here did not pass the Shapiro–Wilk test and therefore was treated as non‐parametric. All results are represented as box plots where the median value is represented by the horizonal line within each box, while the space around the median indicates the interquartile range. Error bars indicate the maximum and minimum values for each group. To determine whether swimming distance (cm) or mean swimming velocity (cm/s) was altered by individual drug treatments and co‐treatments, non‐parametric analysis was performed using either the Mann–Whitney test, or in most cases by using the Kruskal–Wallis test followed by Dunn's multiple comparisons test to determine statistical differences between treatment groups (*p* < 0.05). For the notation of statistical significance, columns which share the same letter(s) of the alphabet are not statistically different from one another. All experiments were replicated four times (*N* = 4).

## 3 RESULTS

### 3.1 Blocking CB1R prevents swimming deficits caused by singular inhibition of MAGL and dual inhibition of FAAH/MAGL

**FIGURE 1:**
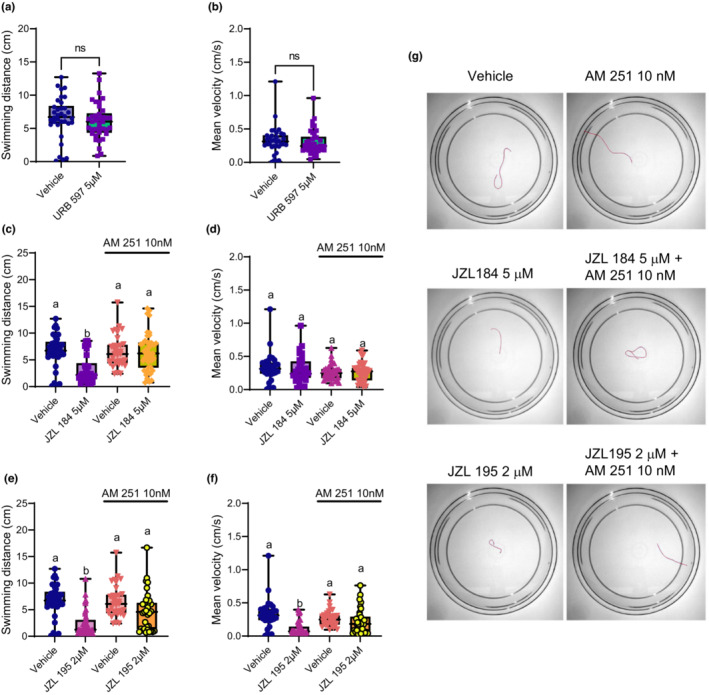
Escape swimming deficits caused by singular MAGL or dual FAAH/MAGL inhibition is rescued by blocking CB1R. Escape swimming in response to an acute touch stimulus was assessed in zebrafish embryos at 2 days post‐fertilization (dpf). The effect of ~24‐h drug treatment on embryonic escape swimming distance (cm) and mean velocity (cm/s) was measured, and mean ranks of treatment groups were compared against each other. In each respective treatment group, the horizontal line within its boxplot represents the median value which is surrounded by the interquartile range, while the error bars represent the maximum and minimum values. (A, B) The effects of 5 μM URB 597 (*n* = 38) on escape swimming are compared relative to the vehicle control (*n* = 34), where ns = not statistically significant (Mann–Whitney test—where statistical significance was determined as *p* < 0.05). (C, D) The effects of the vehicle control (*n* = 34), 5 μM JZL 184 (*n* = 30), 10 nM AM 251 (*n* = 33), and 5 μM JZL 184 in the presence of 10 nM AM 251 (*n* = 43) on escape swimming was compared. (E, F) The effects the vehicle control (*n* = 34), 2 μM JZL 195 (*n* = 39), 10 nM AM 251 (*n* = 33), and 2 μM JZL 195 in the presence of 10 nM AM 251 (*n* = 34) on escape swimming was compared. *N* = 4 experiments with 8–11 animals per experiment for each treatment. (G) Representative tracings of the swimming paths are shown for vehicle, 10 nM AM 251, 5 μM JZL 184, 5 μM JZL 184 + 10 nM AM 251, 2 μM JZL 195, and 2 μM JZL 195 + 10 nM AM 251. For (C–F), columns which share the same letter(s) of the alphabet are not statistically different from one another (Kruskal–Wallis, followed by Dunn's multiple comparisons test—where statistical significance was determined as *p* < 0.05).

It was shown previously that zebrafish embryos exposed to the FAAH and MAGL inhibitors URB 597, JZL 184, and JZL 195 experience deficits in locomotion, and so here we examined the locomotor alterations caused by inhibiting these eCS catabolic enzymes (Khara et al., 2022; Sufian et al., 2021). Because FAAH and MAGL inhibition are linked to increased eCB levels, our investigation began by targeting the canonical cannabinoid receptors: CB1R and CB2R (Griebel et al., 2015; Zou & Kumar, 2018). Singular inhibition of FAAH using URB 597 did not produce any significant alterations to swimming distance, or to swimming velocity, relative to the vehicle control (*p* = 0.175 and 0.218, respectively; Figure 1a,b). Consequently, the rest of our assessments focused on the effects caused by JZL 184 and JZL 195.

Treatment with the MAGL inhibitor JZL 184 caused a significant reduction in swimming distance relative to the vehicle control (*p* < 0.001; Figure 1c). Co‐treatment of JZL 184 with the CB1R antagonist AM 251 prevented the deficits caused by JZL 184 (*p* < 0.001), indicating that blocking CB1R prevents the hypo‐locomotion effects caused by MAGL inhibition. No statistically significant effects on mean swimming velocity were observed from the individual treatment of JZL 184, or the combined JZL 184 + AM 251 treatments (Figure 1d).

Dual inhibition of FAAH/MAGL with JZL 195 caused a significant reduction of swimming distance relative to the vehicle control (*p* < 0.001; Figure 1e). Co‐treatment of JZL 195 with AM 251 partially blocked the effects of JZL 195 (*p* = 0.020). Similarly, JZL 195 caused a similar reduction to mean swimming velocity relative to the vehicle control (*p* < 0.001). AM 251 prevented the effects of JZL 195 on swimming velocity (*p* = 0.015; Figure 1f). This finding demonstrates that blocking CB1R results in recovery from the locomotor deficits caused by FAAH/MAGL inhibition and that CB1R is involved in the motor deficits induced by perturbed eCB signaling.

### 3.2 Swimming deficits caused by FAAH/MAGL suppression are still present when blocking CB2R

**FIGURE 2:**
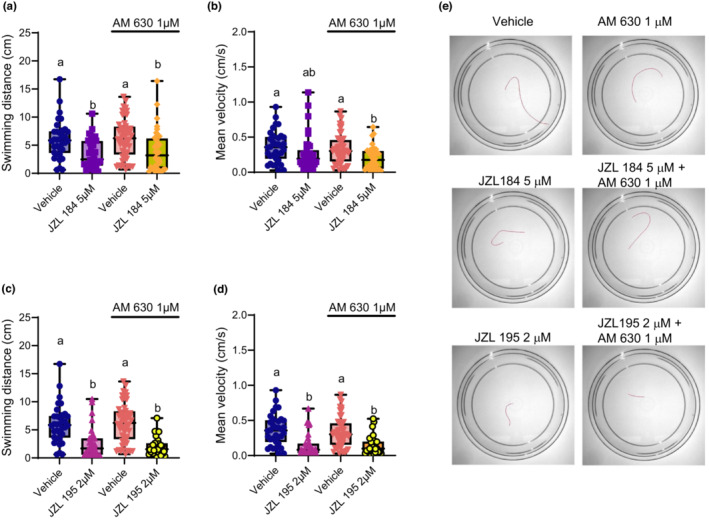
Reductions to swimming performance caused by singular MAGL or dual FAAH/MAGL inhibition are still observed when blocking CB2R. Escape swimming in response to an acute touch stimulus was assessed in zebrafish embryos at 2 days post‐fertilization (dpf). The effect of ~24‐h drug treatment on embryonic escape swimming distance (cm) and mean velocity (cm/s) was measured, and mean ranks of treatment groups were compared against each other. In each respective treatment group, the horizontal line within its boxplot represents the median value which is surrounded by the interquartile range, while the error bars represent the maximum and minimum values. (A, B) The effects of the vehicle control (*n* = 35), 5 μM JZL 184 (*n* = 36), 1 μM AM 630 (*n* = 52), and 5 μM JZL 184 in the presence of 1 μM AM 630 (*n* = 36) on escape swimming was compared. (C, D) The effects of the vehicle control (*n* = 35), 2 μM JZL 195 (*n* = 36), 1 μM AM 630 (*n* = 52), and 2 μM JZL 195 in the presence of 1 μM AM 630 (*n* = 28) on escape swimming was compared. (E) Representative tracings of the swimming paths are shown for vehicle, 1 μM AM 630, 5 μM JZL 184, 5 μM JZL 184 + 1 μM AM 630, 2 μM JZL 195 and 2 μM JZL 195 + 1 μM AM 630. *N* = 4 experiments with 7–13 animals per experiment for each treatment. Columns which share the same letter(s) of the alphabet are not statistically different from one another (Kruskal–Wallis, followed by Dunn's multiple comparisons test—where statistical significance was determined as *p* < 0.05).

Next, we tested the ability of the CB2R antagonist AM 630 to prevent the effects of JZL 184 and JZL 195. Our results indicate that blocking CB2R did not prevent the reductions in swimming distance caused by JZL 184 (*p* > 0.99; Figure 2a). In terms of mean swimming velocity, animals co‐treated with JZL 184 + AM 630, and animals treated with only JZL 184 had similar swimming velocity in their escape responses (*p* > 0.99; Figure 2b). The JZL 184 + AM 630 group did however exhibit a significant reduction in velocity relative to the vehicle control group and the AM 630 control group (*p* = 0.004 and 0.011, respectively).

We observed similar results for animals treated with the dual FAAH/MAGL inhibitor, JZL 195 and AM 630 (*p* > 0.99; Figure 2c). However, we found that the JZL 195‐induced reduction in swimming velocity was not significantly different from the JZL 195 + AM 630 co‐treatment group (*p* > 0.99; Figure 2d) suggesting that motor deficits experienced in 2 dpf embryos caused by eCS perturbation are not mediated through the activity of CB2R.

### 3.3 Blocking the activity of TRP channels prevents locomotor deficits caused by dual FAAH/MAGL inhibition, but not deficits caused by singular MAGL inhibition

Next, we asked if TRP channels were involved in mediating the embryonic motor deficits caused by eCS perturbation. We used the TRP channel blocker AMG 9090 which blocks he activity of TRPA1, TRPV1, and TRPM8 cation channels (Klionsky et al., 2007; Miller et al., 2014).

**FIGURE 3:**
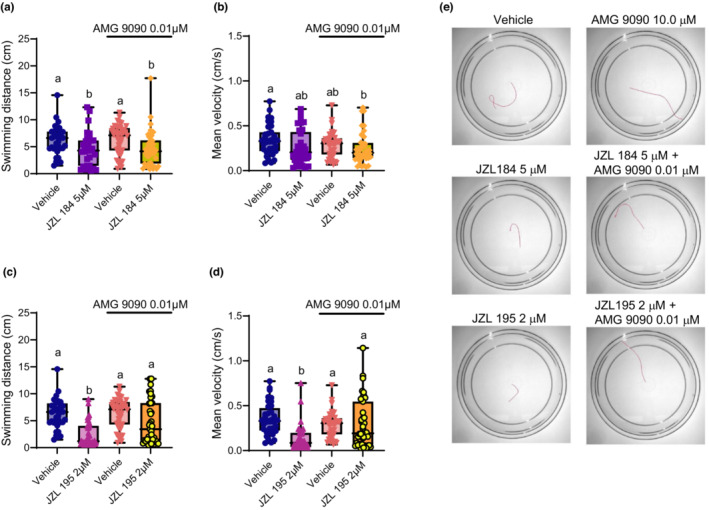
Blocking TRP channels with AMG 9090 prevents the escape swimming deficits caused by dual FAAH/MAGL inhibition, but not deficits caused by singular MAGL inhibition. Escape swimming in response to an acute touch stimulus was assessed in zebrafish embryos at 2 days post‐fertilization (dpf). The effect of ~24‐h drug treatment on embryonic escape swimming distance (cm) and mean velocity (cm/s) was measured, and mean ranks of treatment groups were compared against each other. In each respective treatment group, the horizontal line within its boxplot represents the median value which is surrounded by the interquartile range, while the error bars represent the maximum and minimum values. (A, B) The effects of the vehicle control (*n* = 35), 5 μM JZL 184 (*n* = 33), 0.01 μM AMG 9090 (*n* = 40), and 5 μM JZL 184 in the presence of 0.01 μM AMG 9090 (*n* = 40) on escape swimming was compared. (C, D) The effects the vehicle control (*n* = 35), 2 μM JZL 195 (*n* = 38), 0.01 μM AMG 9090 (*n* = 40), and 2 μM JZL 195 in the presence of 0.01 μM AMG 9090 (*n* = 39) on escape swimming was compared. (E) Representative tracings of the swimming paths are shown for vehicle, 0.01 μM AMG 9090, 5 μM JZL 184, 5 μM JZL 184 + 0.01 μM AMG 9090, 2 μM JZL 195 and 2 μM JZL 195 + 0.01 μM AMG 9090. *N* = 4 experiments with 8–10 animals per experiment for each treatment. Columns which share the same letter(s) of the alphabet are not statistically different from one another (Kruskal–Wallis, followed by Dunn's multiple comparisons test—where statistical significance was determined as *p* < 0.05).

Co‐treatment of JZL 184 with AMG 9090 gave results that were similar to JZL 184 alone on swimming distance (*p* > 0.99; Figure 3a). Similarly, the mean velocity of animals treated with JZL 184 + AMG 9090 was statistically similar to JZL 184 alone (*p* > 0.99) and to the AMG 9090 control group (*p* = 0.747; Figure 3b). These findings suggest that TRP channel inhibition is ineffective in preventing locomotor deficits caused by JZL 184 in 2 dpf embryos.

Interestingly, animals co‐treated with JZL 195 and AMG 9090 experienced a recovery in escape swimming performance. For example, AMG 9090 prevented the effects of JZL 195 on both swimming distance and velocity (*p* < 0.015 and 0.003, respectively; Figure 3c,d) suggesting that TRP channels may play a partial role in mediating the locomotor alterations caused by eCS perturbation.

### 3.4 SMO agonism rescues the alterations to 2 dpf swimming caused by singular MAGL inhibition and partially attenuates the alterations caused by dual FAAH/MAGL inhibition

Since the SHH signaling pathway is critical in neurodevelopment and because it is known to be involved in eCB signaling, we asked if upregulating SHH activity offset the locomotor deficits caused by eCS perturbation (Boa‐Amponsem et al., 2019; Choudhry et al., 2014; Ericson et al., 1996; Khaliullina et al., 2015). To do this we examined the escape swimming performance of 2 dpf embryos that had been exposed to JZL 184 or JZL 195 in combination with the SMO agonist purmorphamine.

**FIGURE 4:**
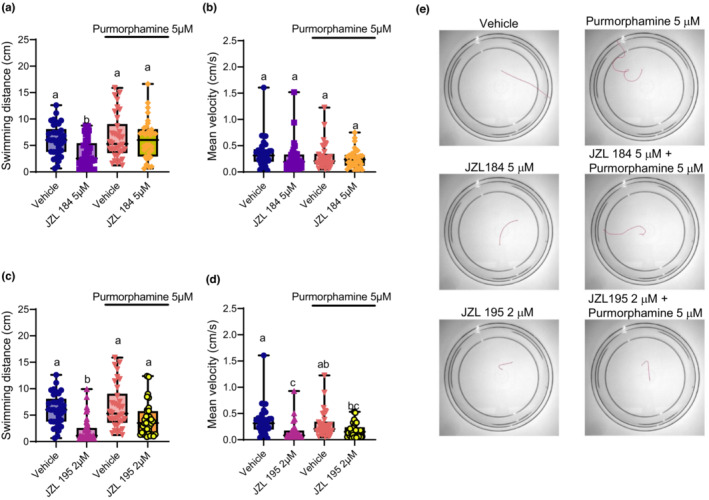
Activation of smoothened rescues locomotor deficits caused by MAGL inhibition and partially rescues escape swimming deficits caused by dual FAAH/MAGL inhibition. Escape swimming in response to an acute touch stimulus was assessed in zebrafish embryos at 2 days post‐fertilization (dpf). The effect of ~24‐h drug treatment on embryonic escape swimming distance (cm) and mean velocity (cm/s) was measured, and mean ranks of treatment groups were compared against each other. In each respective treatment group, the horizontal line within its boxplot represents the median value which is surrounded by the interquartile range, while the error bars represent the maximum and minimum values. (A, B) The effects of the vehicle control (*n* = 34), 5 μM JZL 184 (*n* = 37), 5 μM purmorphamine (*n* = 34), and 5 μM JZL 184 in the presence of 5 μM purmorphamine (*n* = 34) on escape swimming was compared. (C, D) The effects the vehicle control (*n* = 34), 2 μM JZL 195 (*n* = 38), 5 μM purmorphamine (*n* = 34), and 2 μM JZL 195 in the presence of 5 μM purmorphamine (*n* = 33) on escape swimming was compared. (E) Representative tracings of the swimming paths are shown for vehicle, 5 μM Purmorphamine, 5 μM JZL 184, 5 μM JZL 184 + 5 μM Purmorphamine, 2 μM JZL 195 and 2 μM JZL 195 + 5 μM Purmorphamine. *N* = 4 experiments with 8–10 animals per experiment for each treatment. Columns which share the same letter(s) of the alphabet are not statistically different from one another (Kruskal–Wallis, followed by Dunn's multiple comparisons test—where statistical significance was determined as *p* < 0.05).

Co‐treatment of JZL 184 with purmorphamine resulted in an increase in swimming distance, relative to the reduction caused by JZL 184 alone (*p* < 0.007), indicating that purmorphamine was effective in preventing swimming deficits induced by MAGL inhibition (Figure 4a). There was no statistically significant effect of any JZL 184 treatments on mean swimming velocity (*p* > 0.05; Figure 4b).

For the dual inhibitor JZL 195, purmorphamine co‐treatment resulted in a statistically significant increase in swimming distance relative to the distance swam by embryos exposed to JZL 195 alone (*p* = 0.011; Figure 4c). Co‐treatment with purmorphamine did not increase mean swimming velocity in a statistically significant manner relative to JZL 195 alone (*p* = 0.928; Figure 4d). The swimming velocity of animals exposed to JZL 195 + purmorphamine also was not statistically different from the purmorphamine control group (*p* = 0.130). However, mean swimming velocity of the JZL 195 + purmorphamine treatment group was different from the vehicle control in a statistically significant manner (*p* = 0.003). These findings demonstrate a purmorphamine‐associated recovery from deficits in swimming distance caused by JZL 184 and JZL 195. For JZL 195, this recovery is partial in that purmorphamine is capable of restoring swimming distance, but not velocity. Despite this, the observations reported here support the involvement of the SHH signaling pathway in mediating the developmental influence of the eCS on motor development.

## 4 DISCUSSION

In this study, we provide some of the first evidence that highlights the interplay between eCB signaling with TRP channels and the SHH pathway in early vertebrate motor development. Previous reports demonstrate that the eCS catabolic enzymes FAAH and MAGL are critical for normal motor development and that their disruption during embryogenesis alters locomotion (Khara et al., 2022; Boa‐Amponsem et al., 2019; Sufian et al., 2021). Here, we specifically focused on escape swimming behaviors in 2 dpf zebrafish embryos following a touch‐evoked escape response. This particular assessment was chosen because it signifies the emergence of complex motor function and the integration of mechanical sensation to provide a representation of locomotor development, and sensorimotor function (Saint‐Amant & Drapeau, 1998; Sztal et al., 2016). Furthermore, it has already been established in zebrafish that perturbations of the eCS alter the development of escape swimming and lead to deficits in swimming performance (Khara et al., 2022; Ahmed et al., 2018; Amin et al., 2020). Thus, analyzing this in zebrafish is advantageous for studying the interactions of eCB signaling in the development of neuromuscular systems. The present study aimed to further evaluate the mechanisms behind these locomotor alterations and to establish whether non‐canonical eCB signaling mechanisms play a role in mediating eCS‐related locomotor deficits in 2 dpf zebrafish.

Our findings show that hypo‐locomotion caused by inhibiting MAGL and by dual FAAH/MAGL inhibition occurs through a CB1R mechanism, as opposed to a CB2R mechanism. Furthermore, we demonstrate that locomotor deficits caused by dual FAAH/MAGL inhibition are attenuated through blocking TRP channels. Lastly, our findings suggest that SHH signaling is also involved, as SMO activation rescued the swimming impairments caused by inhibition of MAGL and dual inhibition of FAAH and MAGL. Importantly, these results provide a brief overview that signifies the endocannabinoid system's interactions with multiple receptors and signaling mechanisms during embryogenesis, specifically highlighting the importance of non‐canonical cannabinoid receptor mechanisms in motor development.

Regulation of eCB signaling has been implicated in the control of motor activities, and activity of CB1R is critical for the development of reticulospinal neurons involved in locomotor and sensorimotor programs (Rodríguez de Fonseca et al., 1998; Watson et al., 2008). With respect to CB1R in embryonic zebrafish, motor neuron aberrations, and hypo‐locomotion caused by FAAH/MAGL inhibition are partially rescued by AM 251 co‐treatment (Sufian et al., 2021). In rodent models, hypo‐locomotion caused by JZL 184 and JZL 195 were prevented by administration of the CB1R antagonist SR141716A, while another study showed that altered exploratory swimming behaviors caused by JZL 195 was partially restored with SR141716A treatment in zebrafish (Boa‐Amponsem et al., 2019; Seillier et al., 2014). Additionally, consistent with our findings, eCS‐related locomotor alterations do not appear to occur through the CB2R (Sufian et al., 2021). AEA is known to preferentially bind to the CB1R, while 2‐AG is known to have affinity toward both the CB1R and CB2R, which further contextualizes the greater prevalence of CB1R activation in these previous reports, as well as in our study (Zou & Kumar, 2018).

The involvement of TRP channels is not well‐characterized as it pertains to zebrafish motor development, in part because they are mainly recognized as mediators of ionic homeostasis and act as crucial receptor mechanisms in sensory systems (Nilius & Owsianik, 2011). In zebrafish, TRPA1 is abundant in the lateral line neuromast hair cells, serving as mechanosensory receptors (Germanà et al., 2018). In terms of locomotor activity, the presence of these channels likely plays an important role for sensing water motion, thus facilitating functional movement (Moorman, 2001). Our findings provide evidence that there is interplay between TRP channels and eCS signaling in facilitating functional locomotor development. The mechanisms through which TRP channels can interact with eCBs to facilitate functional motor systems remain unclear. However, using whole‐mount in‐situ hybridization of zebrafish, our group has previously demonstrated that *trpv1* expression is detectable as early as 1 dpf within the trigeminal neurons, lateral line, and Rohon‐Beard neurons, indicating that during embryogenesis, these receptor systems are located on key mechanosensory structures involved in motor and sensorimotor activities (McArthur et al., 2020; Son & Ali, 2022). Trigeminal neurons in particular are involved in relaying mechanical stimuli, along with chemical and temperature‐related information across the spinal cord and hindbrain (Pan et al., 2012). Given that eCBs have affinities for TRPA1 and TRPV1, this suggests that a potential consequence of FAAH/MAGL inhibition would be the precocious activation of either trigeminal TRPA1/TRPV1, and activation of lateral line or Rohon‐Bead TRPV1, likely leading to the altered development or defective functionality of these structures (Muller et al., 2019; Watanabe et al., 2003).

Our results indicate that TRP channel blockade was effective in preventing deficits caused by dual FAAH/MAGL inhibition, but not by singular inhibition of MAGL. Although this potentially implicates the roles of AEA and the FAAH enzyme, our findings in this study demonstrate that singular FAAH inhibition does not alter this aspect of locomotion. Likewise, our previous report demonstrates that singular FAAH inhibition using URB 597 does not produce detectable alterations in a variety of different sensorimotor activities (Khara et al., 2022). Although our results suggest a more dominant influence from 2‐AG signaling, AEA's potential role here is still elusive and must be studied further. In terms of dual inhibition by JZL 195, this result may be explained through the greater extent of cannabinoid receptor activation due to the elevation of both AEA and 2‐AG signaling, which potentially relates to a greater extent of eCS‐TRP channel interactions. For instance, in the mouse brain, CB1Rs colocalize with TRPV1 (Cristino et al., 2006). Importantly, this colocalization is detected in the cerebellum, implicating a relation to motor function/coordination. Additionally, CB2Rs and TRPV1 have been shown to colocalize in human dorsal root ganglia (Anand et al., 2008). Thus, because eCB signaling is elevated by JZL 195 which leads to increased activation of cannabinoid receptors, and because of their potential for colocalization, the activated cannabinoid receptors may undergo cross‐talk with TRPV1. Ultimately, due to the upregulated activity of cannabinoid receptors, this cross‐talk may lead to an increased extent of TRPV1 activation. This eCS‐TRP channel interaction may serve as a potential mechanism by which eCBs can influence sensorimotor function and development through TRPV1. Here, in using AMG 9090 to block the activity of TRP channels, this effect may be partially diminished in terms of the activation of TRP channels, however, TRP channel blockade itself was sufficient to rescue the escape swimming deficits in 2 dpf animals. Despite the interpretations proposed here, further embryonic studies must be done to more directly ascertain how eCBs interact with different TRP subtypes to influence motor development.

The SHH pathway recently has been revealed to possess the capacity to interact with cannabinoids (Boa‐Amponsem et al., 2019; Khaliullina et al., 2015). Proximity ligation experiments demonstrated that CB1R and SMO form a heteromer, illustrating the potential for eCB‐SHH interactions (Fish et al., 2019). Both the CB1R and SHH signaling serve as critical components in neural development (Choudhry et al., 2014; Watson et al., 2008). For instance, the CB1R is important for the development of hindbrain reticulospinal neurons in zebrafish, which are crucial for sensorimotor function (Watson et al., 2008). Previous work also demonstrates that perturbations of the eCS in embryonic zebrafish lead to deficits in the development of motor neurons and locomotor activities, and these alterations were demonstrated to be mediated through the CB1R (Ahmed et al., 2018; Sufian et al., 2019; Sufian et al., 2021). Meanwhile, SHH signaling is crucial for neural tube formation and is required for motor neuron differentiation (Ericson et al., 1996). Furthermore, motor recovery in CTB‐saporin‐induced depletion of motor neurons was correlated with increased SHH expression in the lumbar spinal cord of mice (Gulino et al., 2017). Therefore, it is clear that while the eCS and SHH pathways play important roles in the neurobiology of locomotor activities, our findings here suggest that their influences likely converge when it comes to functional locomotor development. In our case, the SMO agonist Purmorphamine was effective in preventing swimming deficits caused by JZL 184 and JZL 195. This indicates that 2‐AG signaling is likely coupled with SMO activity and that this dynamic must be appropriately regulated to facilitate normal motor development. Overall, the recent findings suggesting that SHH signaling is a mechanism of action for cannabinoids support our observations of an eCS‐SMO interaction in the motor development of zebrafish (Boa‐Amponsem et al., 2019; Fish et al., 2019).

Although we have observed the interactions of eCB signaling with multiple different receptor systems here, future studies must also consider the possibility of other candidate receptors and signaling pathways that are capable of interacting with cannabinoids. For example, GPR55 is known to interact with eCBs and phytocannabinoids and is now commonly thought of as a cannabinoid receptor (Amin & Ali, 2019; Lauckner et al., 2008). In terms of developmental signaling, a recent report that used morpholino oligonucleotides has suggested that there is cross‐talk between eCB signaling and fibroblast growth factor (FGF) signaling in the neurobiological development of behavioral processes in zebrafish (Boa‐Amponsem et al., 2020). This study assessed juvenile zebrafish and found that an overexpression of *fgf8* mRNA rescued the behavioral alterations that were caused by embryonic exposure to the CB1R agonist ACEA. Given the locomotor behavioral recoveries that were mediated through certain receptor systems in the current study, it will also be useful to ascertain the involvement of GPR55 and FGF signaling when studying the role of the eCS in neurobiological and motor development.

Despite our current understanding of the endocannabinoid system's role in neurobiological development, the multitude of receptor, and signaling mechanisms through which eCBs interact with remains enigmatic. With the goal of addressing this knowledge gap, we have studied the effects of eCS perturbations in order to outline the involvement of non‐canonical cannabinoid receptor mechanisms in the early locomotor development of embryonic zebrafish. To our knowledge, the findings presented here represent one of the first reports of TRP channel and SHH involvement in eCB‐related motor development. While more information regarding these signaling systems is essential for more precisely mapping out the different interactions of the eCS, the current work establishes a foundation upon which we may begin to unravel the intricacies of eCB signaling during early development.

## ETHICS STATEMENT

This study was conducted in compliance with the Canadian Council on Animal Care (CCAC) guidelines for the humane use of animals for research purposes and in accordance with The International Council for Laboratory Animal Science (ICLAS) Ethical guidelines.
